# Randomized phase-III study of low-dose cytarabine and etoposide + /− all-*trans* retinoic acid in older unfit patients with *NPM1*-mutated acute myeloid leukemia

**DOI:** 10.1038/s41598-023-41964-y

**Published:** 2023-09-08

**Authors:** R. F. Schlenk, D. Weber, J. Krzykalla, T. Kindler, G. Wulf, B. Hertenstein, H. R. Salih, T. Südhoff, J. Krauter, U. Martens, S. Wessendorf, V. Runde, H. J. Tischler, M. Bentz, E. Koller, M. Heuser, F. Thol, A. Benner, A. Ganser, K. Döhner, H. Döhner

**Affiliations:** 1 NCT-Trial Center, National Center of Tumor Diseases, Heidelberg University Hospital and German Cancer Research Center, Im Neuenheimer Feld 130.3, 69120 Heidelberg, Germany; 2 Department of Internal Medicine V, Heidelberg University Hospital, Heidelberg, Germany; 3 Department of Internal Medicine III, University Hospital of Ulm, Ulm, Germany; 4 Division of Biostatistics, German Cancer Research Center Heidelberg, Heidelberg, Germany; 5 Department of Hematology, Medical Oncology and Pneumology, University Medical Center Mainz, Mainz, Germany; 6 Department of Hematology and Oncology, University Hospital of Göttingen, Göttingen, Germany; 7 Department of Hematology and Oncology, Klinikum Bremen Mitte, Bremen, Germany; 8 Department of Hematology and Oncology, Eberhard-Karls University, Tübingen, Germany; 9 Department of Hematology and Oncology, Klinikum Passau, Passau, Germany; 10 Department Hematology and Oncology, Braunschweig Municipal Hospital, Braunschweig, Germany; 11 Department of Hematology and Oncology, Klinikum am Gesundbrunnen, Heilbronn, Germany; 12 Department of Hematology and Oncology, Klinikum Esslingen, Esslingen, Germany; 13 Department of Hematology/Oncology, Wilhelm-Anton Hospital Goch, Goch, Germany; 14 Department of Hematology and Oncology, University Hospital of Minden, Minden, Germany; 15 Department of Hematology and Oncology, Städtisches Klinikum Karlsruhe, Karlsruhe, Germany; 16 Department of Internal Medicine III, Hanuschkrankenhaus Wien, Wien, Austria; 17 Department of Hematology, Hemostasis, Oncology and Stem Cell Transplantation, Hannover Medical School, Hannover, Germany

**Keywords:** Cancer, Oncology

## Abstract

The aim of this randomized clinical trial was to evaluate the impact of all-*trans* retinoic acid (ATRA) in combination with non-intensive chemotherapy in older unfit patients (> 60 years) with newly diagnosed *NPM1*-mutated acute myeloid leukemia. Patients were randomized (1:1) to low-dose chemotherapy with or without open-label ATRA 45 mg/m^2^, days 8–28; the dose of ATRA was reduced to 45 mg/m^2^, days 8–10 and 15 mg/m^2^, days 11–28 after 75 patients due to toxicity. Up to 6 cycles of cytarabine 20 mg/day s.c., bid, days 1–7 and etoposide 100 mg/day, p.o. or i.v., days 1–3 with (ATRA) or without ATRA (CONTROL) were intended. The primary endpoint was overall survival (OS). Between May 2011 and September 2016, 144 patients (median age, 77 years; range, 64–92 years) were randomized (72, CONTROL; 72, ATRA). Baseline characteristics were balanced between the two study arms. The median number of treatment cycles was 2 in ATRA and 2.5 in CONTROL. OS was significantly shorter in the ATRA compared to the CONTROL arm (*p* = 0.023; median OS: 5 months versus 9.2 months, 2-years OS rate: 7% versus 10%, respectively). Rates of CR/CRi were not different between treatment arms; infections were more common in ATRA beyond treatment cycle one. The addition of ATRA to low-dose cytarabine plus etoposide in an older, unfit patient population was not beneficial, but rather led to an inferior outcome.

The clinical trial is registered at clinicaltrialsregister.eu (EudraCT Number: 2010-023409-37, first posted 14/12/2010).

## Introduction

Mutations in the Nucleophosmin-1 (*NPM1*) gene occur in ∼ 20 to 33% of adult patients with acute myeloid leukemia (AML)^1–4^ and are present in all age groups and still about one fifth of patients above the age of 70 years exhibit *NPM1* mutation^4^. AML with mutated *NPM1* is recognized as a disease entity in the WHO classification^5^ and clinical trials focusing on this disease entity are emerging^6,7^.

All-*trans* retinoic acid (ATRA) in combination with chemotherapy or arsenic trioxide has revolutionized the treatment of acute promyelocytic leukemia (APL)^8,9^. Furthermore, preclinical and clinical studies also provided a rationale for the use of ATRA in non-APL AML^10–12^ and in particular in AML with mutated *NPM1*^13–18^. However, results from large randomized studies evaluating ATRA in combination with intensive but also non-intensive chemotherapy have been contradictory^12,17–24^.

In three studies performed by the British Medical Research Council (MRC), one in younger patients receiving intensive first-line treatment (MRC AML12, n = 1097)^22^, one in medically unfit patients (MRC AML14, n = 207)^23^, and one in high-risk, refractory or relapsed patients (MRC AML-HR, n = 362)^24^, consistently negative results were reported overall and in genetic subgroups^20^. In the study by Estey et al. of 215 patients with high-risk myelodysplastic syndrome or AML older than 71 years, there was no effect of ATRA in multivariable analysis, but a significantly better overall survival was found in univariable analyses for patients treated in the ATRA arms^21^. In all these trials, ATRA was started simultaneously or before initiation of chemotherapy^21–24^. In contrast, in the trials conducted by the German-Austrian Study Group (AMLSG), ATRA was started at the end of chemotherapy^6,17–19^ in accordance with the in vitro data^10,11^. In these trials, older patients randomized to the ATRA arm had a significantly higher complete remission (CR) rate, better event-free (EFS) and overall survival (OS)^17^, whereas in younger patients only per protocol but not intention-to-treat analysis revealed a better EFS and OS for patients receiving ATRA as adjunct to intensive chemotherapy^19^. Similar results were found in subgroup analysis of *NPM1*-mutated AML indicating a benefit in older patients on an intention to treat basis and in younger patients only in a per protocol analysis^18,19^.

In 2011, the AMLSG initiated the randomized AMLSG 15–10 trial in older patients with newly diagnosed AML with *NPM1* mutation not fit for intensive chemotherapy evaluating ATRA in combination with low-dose cytarabine plus etoposide. Here, we report the results of the upfront randomization for ATRA in 144 older adult patients.

## Patients and methods

### Patients

Screening for *NPM1* mutations was performed in patients with newly diagnosed AML within the AMLSG BiO study^4^. Between May 2011 and September 2016, 144 patients were enrolled in the AMLSG 15-10 study. Patients aged > 60 years with newly diagnosed AML including de novo AML, secondary AML with a preceding history of myelodysplastic or myeloproliferative disorder (sAML), and therapy-related AML following treatment of a primary malignancy (tAML), as defined by the WHO 2008 classification were eligible for the trial^25^. Patients not eligible for intensive chemotherapy were included and criteria for unfitness were age ≥ 75 years, hematopoietic cell transplantation-comorbidity index (HCT-CI) > 2 and/or patient decision. Patients with the following disease entities were excluded: AML with t(8;21)(q22;q22.1), *RUNX1*::*RUNX1T1*; AML with inv(16)(p13.1q22) or t(16;16)(p13.1;q22), *CBFB*::*MYH11*; AML with t(15;17)(q24.1;q21.2), *PML*::*RARA* (or other translocations involving *RARA*); AML with t(9;11)(p21.3;q23.3), *MLLT3*::*KMT2A* (or other translocations involving *KMT2A*); AML with t(6;9)(p23.3;q34.1), *DEK*::*NUP214*; AML with inv(3)(q21.3q26.2) or t(3;3)(q21.3;q26.2), *GATA2*, *MECOM(EVI1)*. Furthermore, patients with concomitant renal (creatinine > 1.5 × upper normal serum level), liver (bilirubin, AST or AP > 2.5 × upper normal serum level) or cardiac dysfunction (New York Heart Association III/IV), uncontrolled infectious disease, primary coagulation disturbance or performance status (ECOG) > 2 were excluded. Written informed consent was obtained from all patients. The protocol was approved by the lead Ethics Review Committee (Ethikkommission der Universität Ulm, Ulm, Germany) and registered at clinicaltrialsregister.eu (EudraCT Number: 2010-023409-37) and clinicaltrials.gov (NCT01237808, first posted 10/11/2010) and methods were performed in accordance with the relevant guidelines and regulations.

### Cyto- and molecular genetics

Chromosome banding analysis was performed centrally in the two AMLSG Laboratories for Cytogenetics (Hannover, Ulm). Leukemia samples were analyzed for mutations in *NPM1* and *FLT3* (ITDs, and tyrosine kinase domain [TKD] mutations at codons D835/I836)^2,3,26^.

### Study design

Patients were randomized (1:1) to receive low-dose chemotherapy with or without ATRA. In the first cycle patients received cytarabine 20 mg/day, s.c., bid, days 1–7, and etoposide or etoposidphosphate 50 mg/m^2^/day, continuously i.v., days 1–3; for cycles 2 to 6 the etoposide dose was increased to 100 mg/day, p.o. or i.v. (over 1 h), days 1–3. From May 2011 to March 2014, ATRA was given at a dose of 45 mg/m^2^/day p.o., days 8–28 with or shortly after meals distributed over 3 doses per day. After an interim safety analysis in March 2014, the dose of ATRA was reduced to 45 mg/m^2^ day 8–10, followed by 15 mg/m^2^ day 11–28 due to an increased frequency of toxicities, in particular infections, and deaths observed in the ATRA arm compared to the control arm.

### Definition of response criteria, survival endpoints and hematologic recovery

In accordance with standard criteria, CR was defined as less than 5% bone marrow blasts, an absolute neutrophil count of > 1.0 G/L or higher, a platelet count of 100 G/L or higher, no blasts in the peripheral blood, and no extramedullary leukemia; CR with incomplete blood count recovery (CRi) was defined as CR except for residual neutropenia (neutrophils < 1.0 G/L) or thrombocytopenia (platelets < 100 G/L)^27^. Relapse was defined as more than 5% bone marrow blasts unrelated to recovery from the preceding course of chemotherapy or new extramedullary leukemia in patients with previously documented CR.

EFS and OS were defined as recommended^27^. Toxicities were defined and graded according to the National Cancer Institute (NCI) Common Toxicity Criteria, version 3.0.

### Randomization, sample size calculation, and statistical analysis

Randomization was performed at the AMLSG Clinical Trials Office, two-arm in a ratio of 1:1 using the minimization approach of Pocock (Biometrics 1975) for the factors *FLT3*-ITD and HCT-CI score. After the randomization in the AMLSG Clinical Trials Office, the result of the randomization and the definite patient identification number (patient-ID) were marked on the registration confirmation form, which was returned by Fax to the registering physician and the local investigator.

The sample size calculation was based on data from the British MRC 14 trial^23^ leading to an expected survival probability after 2 years within the control arm of the proposed study of 10%. An improvement by 15% to a 2-year survival probability of 25% in the ATRA arm was defined as clinically relevant. Type I and II errors were fixed at 5% and 20%, respectively. This leaded to a sample size of 144 patients to be randomized. Trial duration was conservatively assumed with a 5-year accrual time and a minimum of 2 years of follow-up period. A drop-out rate of 5% in each arm was assumed for sample size calculation. Sample size estimation was done according to Lachin and Foulkes (1986) using PASS 2008 (NCSS Kaysville, USA).

Pairwise comparisons between patient subgroups were performed by the Mann–Whitney or Kruskal Wallis test for continuous variables and by Fisher’s exact test for categorical variables.

The analysis was performed on an ITT basis according to initial randomization. The primary endpoint of the study was OS; secondary endpoints were event-free survival (EFS), response to therapy (CR/CRi), cumulative incidences of relapse (CIR) and death in CR/CRi (CID) and therapy-related toxicity. The median duration of follow-up was calculated by the reverse Kaplan–Meier estimate^28^; the Kaplan–Meier method was used to estimate the distributions of OS and EFS. Survival distributions were compared using the log-rank test. Multivariable Cox regression models were used to evaluate prognostic variables and the following variables were evaluated: WBC (log10 transformed), age, randomization (CONTROL, ATRA), *FLT3-*ITD, HCT-CI score (≤ 2 versus > 2), ECOG performance status (0–1 versus > 1). A multivariable logistic regression model was applied to investigate the influence of covariates on response to therapy (same covariates as listed above).

CIR and CID were assessed using the time from achievement of CR/CRi until relapse or death in CR/CRi. CIR and CID and their standard errors were estimated according to the method of Aalen and Johansen^29^ and formal statistical comparison of the incidences was done using the test by Gray^30^. A cause-specific Cox model was used for the time to relapse with death in CR as competing event, including the same covariates as for OS and EFS. Fitting the analogous model for time to death in CR with relapse as a competing event was not possible due to low number of events.

Comparisons regarding safety endpoints were performed using Barnard’s test.

All statistical analyses were performed with the statistical software environment R, version 4.3.1, using the R packages survival, version 3.2-13, and cmprsk, version 2.2-11^31^.

## Results

### Patients and baseline characteristics

**Figure 1:**
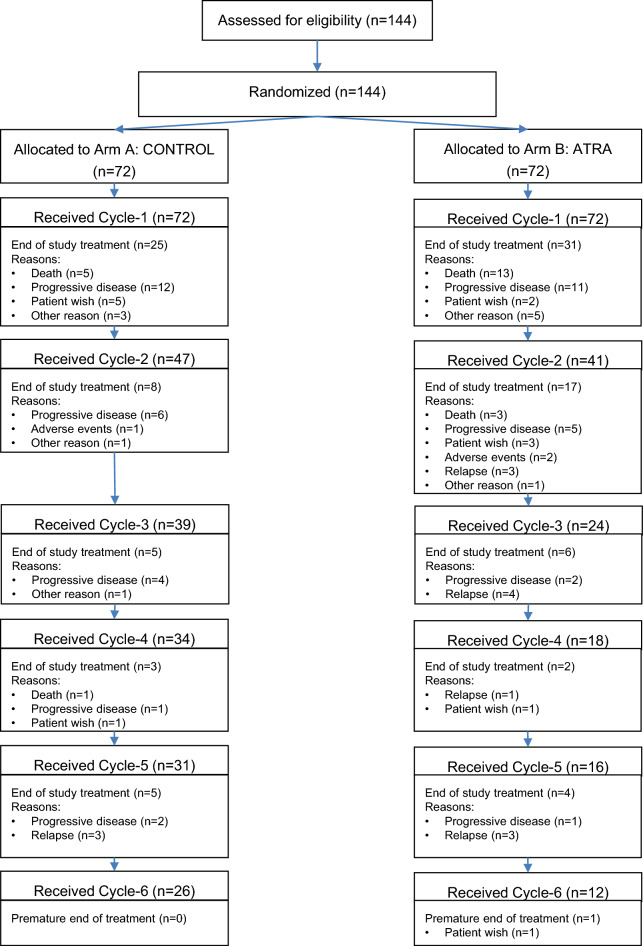
Flow-chart on study conduct. Flow-chart showing enrollment, program completion and/or drop-out according to the randomization result.

A total of 144 patients were randomized, 72 patients to ATRA and 72 to CONTROL. Recruitment started on May 11th 2011, last patient was enrolled on September 14th 2016 with no interruption of enrolment and a minimum follow-up after last patient in of 2 years. Patient demographics and presenting laboratory and genetic characteristics were balanced between the two treatment arms (Table 1). The trial flow is summarized in the diagram according to CONSORT statement in Fig. 1.

**Table 1:** Patient and disease characteristics according to randomization.

	All patients (n = 144)	Standard-arm (n = 72)	ATRA-Arm (n = 72)	*p* value
Age in years, Median (Range)	76.8 (63.8, 91.8)	77.1 (67.7–86.7)	76.4 (63.8, 91.8)	0.63
Male sex, No. (%)	74 (51.4)	39 (54.2)	35 (48.6)	0.37
WHO performance status, No. (%)				
0–1	89 (61.8)	44 (61.1)	45 (62.6)	0.94
> 1	55 (48.2)	28 (38.9)	27 (37.4)	
HCTCI Score, No. (%)				
0–2	72 (50)	36 (50)	36 (50)	0.99
> 2	72 (50)	36 (50)	36 (50)	
Type of AML, No. (%)				
De novo AML	126 (87.5)	63 (87.5)	63 (87.5)	1.00
s-AML	5 (3.5)	3 (4)	2 (3)	
t-AML	13 (9)	6 (8.5)	7 (9.5)	
WBC, 10^9^/L				
Median (Range)	20.4 (0.4–355)	21.4 (0.4–355)	20.3 (0.5–287)	0.21
Missing	1	0	1	
Hemoglobin, g/dL				
Median (Range)	9.1 (5.2–13.2)	8.95 (5.2–13.0)	9.22 (5.2–13.2)	0.72
Missing	1	0	1	
Platelets, 10^9^/L				
Median (Range)	66 (4–494)	71 (7–494)	63 (4–289)	0.16
Missing	1	0	1	
*Bone marrow blasts, %				
Median (Range)	80 (5–100)	80 (25–100)	80 (5–100)	0.57
Missing	17	10	7	
*Peripheral blood blasts, %				
Median (Range)	28.5 (0–98)	22 (0–95)	32 (0–98)	0.93
Missing	6	4	2	
LDH, U/l				
Median (Range)	398 (155–2110)	388 (155–1660)	415 (160–2110)	0.17
Missing	0	0	0	
Cytogenetics				
Normal karyotype, n (%)	111 (78.2)	55 (77.5)	56 (78.9)	0.92
Missing	2	1	1	
*FLT3*-ITD, n (%)	39 (27.1)	18 (25)	21 (29.2)	0.71
Missing	0	0	0	
*FLT3*-TKD, n (%)	17 (11.8)	9 (12.5)	8 (11.1)	0.99
Missing	0	0	0	

*ATRA* All-*trans* Retinoic Acid, *WBC* white blood cells, *LDH* lactate dehydrogenase, *s-AML* AML after previous myelodysplastic syndrome or myeloproliferative neoplasm, *t-AML* therapy-related AML, *FLT3-ITD* FMS-like tyrosine kinase 3 gene internal tandem duplication, *FLT3-TKD* FMS-like tyrosine kinase 3 gene tyrosine kinase domain mutation, *HCT-CI* hematopoietic cell transplantation—comorbidity index, *WHO* World Health Organisation.

*In case of bone marrow blasts < 20%, diagnosis of AML was established based on extramedullary disease or peripheral blood blasts > 20%.

### Applied Treatment and Response

After randomization all 144 patients received the first treatment cycle (n = 72 CONTROL, n = 72 ATRA). Overall, 254 treatment cycles were applied in CONTROL and 177 in ATRA (*p* < 0.001), the median number of cycles was 2.5 in CONTROL compared to 2 in ATRA. Only 38 patients (26%) received the intended 6 cycles of therapy, 26 (33%) in CONTROL and 12 (18%) in ATRA.

Best responses (CR/CRi) were achieved in 26 of 72 (36.1%) in CONTROL and in 24 of 72 (33.3%) patients in ATRA (*p* = 0.86) in median after 2.5 cycles (range, 1–6) in CONTROL and after 2.0 cycles (range, 1–5) in ATRA. A logistic regression model including treatment arm, ECOG status, HCT-CI risk score, age, WBC, and *FLT3*-ITD status did not reveal significant prognostic markers***.*** During the first treatment cycle 17 patients died, n = 6 in CONTROL and n = 11 in ATRA (*p* = 0.30).

### Survival analyses

Estimated median follow-up for survival was 29.8 months (95% CI, 25.4-Inf) without difference between the treatment arms (*p* = 0.58). Of 144 randomized patients 130 died.

**Figure 2:**
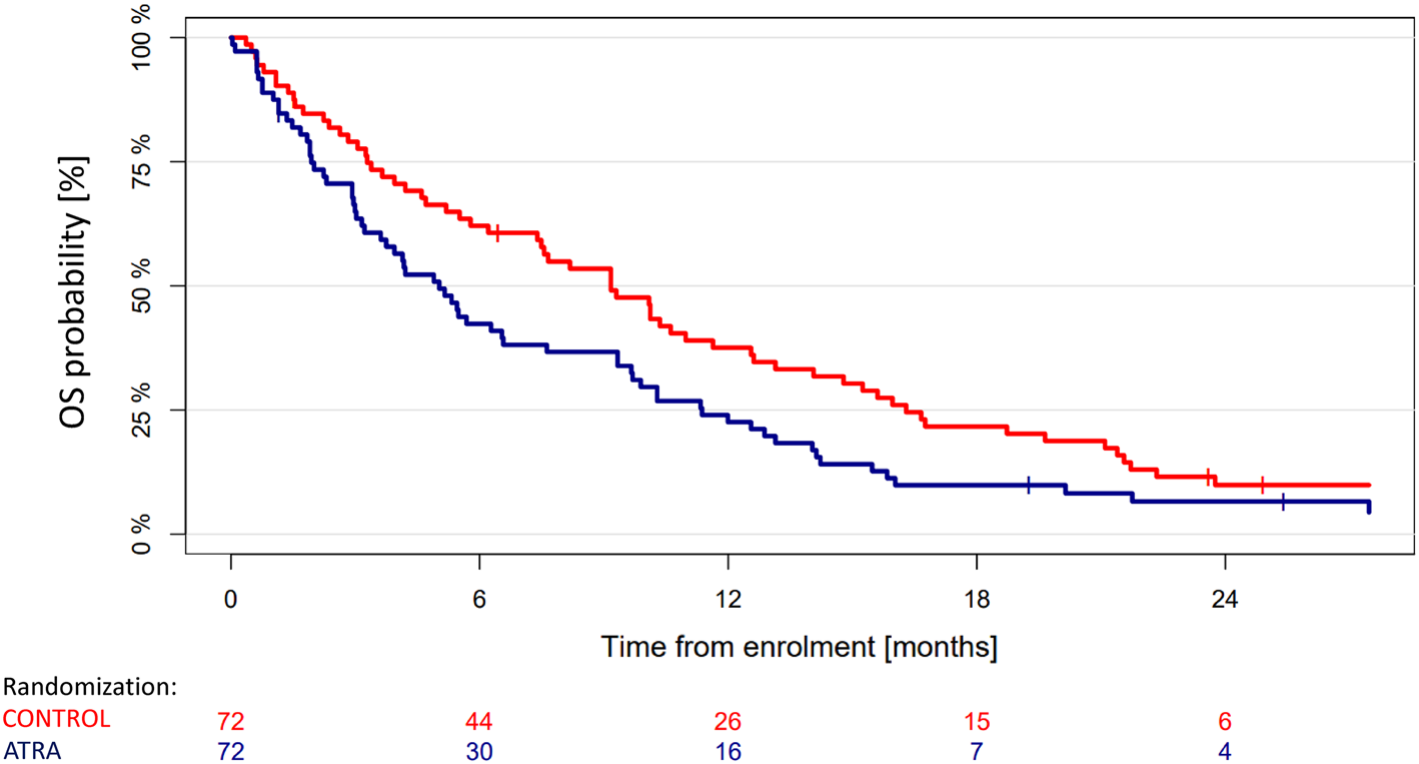
Survival analyses according to randomization according to intention-to-treat analysis. Median survival in ATRA and Control were 5.0 and 9.2 months, respectively (log-rank test, *p* = 0.023).

The log-rank test on an ITT basis for the primary endpoint OS revealed a significantly inferior survival for patients in the ATRA compared to patients in the CONTROL arm (*p* = 0.023, Fig. 2). Median OS times, 1 and 2-years OS rates were 9.2 and 5 months, 38% and 23%, 10% and 7% in CONTROL and ATRA, respectively. Multivariable analyses for OS revealed treatment with ATRA, HCT-CI Score > 2, higher WBC, and older age as significant unfavorable factors (Table 2). No significant difference in EFS was observed between the treatment arms. Of 50 patients achieving a CR/CRi, 45 patients relapsed and 2 patients in ATRA died in CR/CRi. There was no significant difference in CIR (*p* = 0.65) and CID (*p* = 0.14) between the treatment arms. Multivariable (cause-specific) Cox models did not reveal a significant effect of the addition of ATRA on EFS or CIR either. However, results for these endpoints do not provide further insight due to the low response rate.

**Table 2:** Cox regression model on the endpoint overall survival.

	HR	95% CI	*p* value
Treatment Arm—ATRA	1.42	0.99–2.03	0.056
ECOG performance status > 1	1.14	0.78–1.66	0.501
HCTCI risk category > 2	1.52	1.05–2.20	0.025
Age (10 year difference)	1.59	1.10–2.29	0.013
WBC (ten-fold increase)	1.50	1.10–2.05	0.011
*FLT3*-ITD	1.18	0.79–1.77	0.417

*ATRA* all-trans retinoic acid, *ECOG* Eastern Cooperative Oncology Group, *HCTCI* hematopoietic cell transplantation comorbidity index, *WBC* white blood count, FLT3-ITD fms-like tyrosine kinase internal tandem duplication.

### Toxicity

Rates of early/hypoplastic death occurring during the first two treatment cycles did not differ significantly between arms but were with 18.1% in ATRA numerically higher compared to 8.5% in CONTROL (*p* = 0.09).

Infections analyzed on an as treated basis during the first treatment cycle occurred in 59% of patients with no difference between treatment arms (*p* = 0.73). In contrast, starting from treatment cycle 2 rates of infections were higher in ATRA compared to CONTROL (cycle 2, 59% vs. 32%, respectively). Whereas for cycle 2, this difference was statistically significant (*p* = 0.01), no significant difference was observed for treatment cycles after cycle 2. During the first treatment cycle no difference with regard to frequency of adverse event was identified between treatment arms although cardiac events were more frequent (*p* = 0.06) in ATRA (Table 3).

**Table 3:** Treatment-related adverse event occurring during the first cycle according to treatment arm and CTCAE category.

	Standard-arm (n = 78) < grade 3	≥ grade 3	ATRA-Arm (n = 66) < grade 3	≥ grade 3
Cardiac Arrhythmia, no	1	0	0	1
Cardiac general, no	1	1	6	1
coagulation, no	1	0	1	0
Constitutional Symptoms, no	28	3	24	2
Dermatology/skin, no	9	0	7	1
Endocrine, no	0	0	1	0
Gastrointestinal, no	48	4	42	6
Hemorrhage/bleeding, no	9	1	10	2
Hepatobiliary/pancreas, no	2	0	0	0
infection, no	15	25	13	27
Lymphatics, no	2	0	6	0
Metabolic/laboratory, No	22	5	26	7
Neurology, no	9	0	5	1
Ocular/visual, no	0	0	2	0
pain, no	11	2	16	2
Pulmonary/upper respiratory, no	0	0	8	5
Renal/genitourinary, no	6	5	6	3
Syndromes, no	1	1	0	0
Vascular, no	1	0	1	1

*CTCAE* Common terminology criteria for adverse events, *ATRA* All-*trans* retinoic acid.

## Discussion

We previously reported that ATRA given in combination with intensive chemotherapy improves survival in patients 61 years and older with newly diagnosed AML^17^ and in particularly in AML with mutated *NPM1*^18^. The objective of the trial reported here was to perform a confirmatory study in an older patient population not fit for intensive chemotherapy exhibiting mutated *NPM1*. The chemotherapy backbone consisted of low-dose cytarabine^23^ in combination with etoposide^32^. Etoposide was added based on reports indicating a specific clinical efficacy of etoposide in myelomonocytic and monoblastic AML^33,34^ and the observation that patients exhibiting a normal karyotype benefit from induction therapy including etoposide in terms of CR rate^35^. Based on the early preclinical data, we decided to start ATRA at day 8, that is, after all cytotoxic drugs were administered^10,11^. The initial dose of ATRA (45 mg/m^2^, days 8–28) had to be reduced (45 mg/m^2^, days 8–10; 15 mg/m^2^, days 11–28) after 75 patients due to an increased toxicity, in particular infections.

Our results show that the addition of ATRA to low-dose cytarabine plus etoposide in an older, unfit patient population was not beneficial, but rather led to an inferior outcome. OS was significantly shorter in ATRA compared to CONTROL (median OS of 5 vs 9 months; *p* = 0.023). A possible explanation could be the cumulative effect of a higher early/hypoplastic rate, an increased rate of infectious complications by the addition of ATRA which was still present after dose reduction of ATRA in the second half of the trial (*p* = 0.90), and a higher rate of cardiac events in ATRA leading to the observed inferior outcome. Furthermore, the addition of etoposide to standard low-dose cytarabine in combination with ATRA might have increased susceptibility to infectious complications, particularly beyond cycle 1, by extensive mucosal toxicity in this very vulnerable older unfit patient population. Of note, extensive mucocutaneous changes leading to early termination of ATRA treatment was reported in a study of lung cancer patients receiving all-trans-retinoic acid plus cisplatin and etoposide^36^.

Two randomized studies have previously been conducted evaluating ATRA in combination with a standard of care backbone in older unfit patients, the MRC AML14 trial with low-dose cytarabine^23^, and the DECIDER trial with decitabine^12^. In the MRC AML 14 trial the addition of ATRA had no effect on all clinical endpoints, CR rate (*p* = 0.30), survival (*p* = 0.60), as well as analyzed toxicities^23^. In contrast, in the DECIDER trial the addition of ATRA to decitabine resulted in an in trend higher CR rate (*p* = 0.06) and a significantly improved survival (*p* = 0.006) with again no differences in toxicity^12^. Based on these results and the trial presented here one may speculate that there is an additive beneficial effect of ATRA with hypomethylating agents but not with conventional low dose cytotoxic drugs.

Although, the preclinical rationale for examining the addition of ATRA in AML was attractive we were not able to show consistently a beneficial effect across different patient populations^6,17–19^. However, the preclinical rationale as well as recently published clinical data underlines the effect of targeted BCL-2 inhibition in AML^37^ and at least based on data of a cohort study in AML with mutated *NPM1*^38^. Furthermore, promising data support that *NPM1*-mutated AMLs are sensitive to chromatin complex inhibition via targeting menin^39^. In line, patient-derived xenograft models of *NPM1*-mutated AML showed promising results with the menin inhibitor VTP50469^40^ and first data of a subsequent phase I study demonstrated single agent activity also in patients with *NPM1*-mutated AML^41^. Furthermore, first clinical data of the oral spleen tyrosine kinase (SYK) inhibitor entospletinib given in combination with intensive chemotherapy in *NPM1*-mutated AML showed promising activity^42^ and triggered a double-blinded randomized phase-III evaluation (ClinicalTrials.gov Identifier: NCT05020665). In conclusion, ATRA in combination with non-intensive chemotherapy as used in our study led to increased toxicity and inferior OS in patients not fit for intensive chemotherapy with newly diagnosed *NPM1*-mutated AML.

## Data Availability

The datasets used and/or analyzed during the current study are available from the last author on reasonable request.
